# The Impact of Nutritional Supplements on Sarcopenia: A Systematic Review and Meta-Analysis

**DOI:** 10.7759/cureus.88459

**Published:** 2025-07-21

**Authors:** Usman Mansoor, Donna Edano, Maaza Usman, Usman Habib

**Affiliations:** 1 Pediatric Emergency Medicine, Al Jalila Children's Speciality Hospital, Dubai, ARE; 2 Emergency Medicine, St. Peter's Hospital, Chertsey, GBR; 3 Hospital-Based Medicine, Western Health Victoria, Melbourne, AUS; 4 Acute Medicine, Frimley Park Hospital, Frimley, GBR

**Keywords:** aging, nutrient supplements, omega 3, physical function, protein, sarcopenia

## Abstract

Sarcopenia is characterized by progressive loss of muscle mass, strength, and function, and poses a major risk for permanent disability and poor quality of life in elderly patients. Nutritional supplements have been proposed as a potential intervention; however, findings in the literature have been inconsistent, necessitating a comprehensive systematic review and meta-analysis. Therefore, the present review aimed to assess the impact of nutritional supplements on the progression and management of sarcopenia-associated problems, such as muscle mass, strength, and function.

A comprehensive literature search was performed on different electronic databases, such as PubMed, Scopus, ScienceDirect, The Cochrane Library, and Google Scholar. Studies assessing the impact of nutritional supplements on muscle mass and strength functions in sarcopenia patients were included. Narrative synthesis was performed for the presentation of the general characteristics of studies, interventions, and outcomes, while meta-analysis was performed using the random effect model via RevMan 5.4 at the significance level of 0.05. Funnel plots were used for the interpretation of publication bias, methodological quality assessment of randomized controlled trials (RCTs) was performed using the Cochrane Risk of Bias-2.0 (RoB 2) assessment tool, and certainty of evidence using the GRADE (Grading, Reporting, Assessment, Development, and Evaluation) framework.

After screening, 28 studies were included in the review, which focused on nutritional supplements containing protein, amino acids, vitamin D, creatine, omega-3, vitamin B12, zinc, magnesium, and other nutrients. These supplements demonstrated significant differences in improving handgrip strength [std. mean difference (MD): -0.10, 95% confidence interval (CI): -0.21 to 0.00, p=0.05, I^2^=0%], skeletal muscle mass index [std. MD: 0.29 (95% CI: 0.04 to 0.53), p=0.02, I^2^=0%], total fat mass [std. MD: 0.21 (95% CI: 0.01 to 0.41) p=0.04, I^2^=5%]. In contrast, a non-significant difference was observed in skeletal muscle mass [std. MD: 0.16 (95% CI: -0.02 to 0.33) p=0.08, I^2^=0%], appendicular lean mass (std. MD: -0.03 (95% CI: -0.22 to 0.16) p=0.76, I^2^=0%], gait speed [std. MD: 0.01 (95% CI: -0.23 to 0.21) p=0.95, I^2^=65%], and adverse events odds ratio (OR): 1.08 (95% CI: 0.80-1.45) p=0.60, I^2^=0%].

No publication bias was observed, and methodologically, most of the studies were found to have a low RoB, except for five RCTs, which had some concerns in the randomization process. Outcomes, like handgrip strength, skeletal muscle mass index, and adverse events, showed a high certainty of evidence. The skeletal muscle and appendicular lean mass had a moderate certainty of evidence, and gait speed had a low certainty of evidence. This study indicates that nutritional supplements demonstrated potential in improving muscle strength. However, further long-term, multicenter, and longitudinal studies are required to validate these findings.

## Introduction and background

Sarcopenia refers to the progressive and accelerated process of loss of skeletal muscle mass and function associated with advancing age, which affects mobility and leads to impaired physical functions as well as increased risk of adverse events, such as fractures, falls, and premature mortality [1]. Sarcopenia significantly affects the quality of life among the elderly, leading to increased economic burden associated with follow-up medical care [2]. Globally, its prevalence rate is 10% in females and 10% in males, which is lower in Asian communities than in non-Asian individuals of both genders [3]. Moreover, the lowest and highest prevalence is observed in Europe and Oceania, ranging from 10%-27% in those aged ≥60 years and 8%-36% in those <60 years [2].

From a pathophysiological context, sarcopenia is characterized by perturbations ranging from subcellular processes within skeletal myocytes to environmental and social factors [4,5]. Mainly, the pathophysiology of sarcopenia is linked to genetic and environmental factors. Genetic factors include growth factors, gene expression of metabolic and structural proteins, hormones, and inflammatory cytokines, while environmental factors, including chronic diseases, physical inactivity, smoking, sleep disturbance, and alcohol consumption, are well-established [5]. It is also influenced by lifestyle factors, including smoking, malnutrition, diabetes, extreme sleep duration, and other contemporaneous risk factors that exert their impacts during life. However, age is considered the most contributing and significant risk factor, and with advancing age, the chances of sarcopenia will increase. In addition, body weight, total body fat, and lean mass also have a significant association with sarcopenia [6].

These diverse factors are responsible for and significantly contribute to the loss of muscles, including decreased numbers of motor units, neuromuscular junction dysfunction, insulin resistance, inflammation, oxidative stress, and mitochondrial dysfunctions [7-11]. Often, a decline in muscle mass is associated with age because it is considered a normal process of life [12]. In addition to age, malnutrition is another important contributing factor associated with a decline in muscle mass and observed in almost one-fourth of hospitalized elderly patients [13,14]. Malnutrition is frequently seen in older people, with increased functional challenges, morbidities, and mortality.

Both sarcopenia and malnutrition have many similar pathophysiological components, like a low inflammatory state [15]. Several epidemiological studies suggest that chronic malnutrition, poor quality diet, and physical inactivity significantly contribute to sarcopenia and are also associated with a higher risk of mortality in older people. Moreover, sarcopenia is further associated with a wide range of adverse health-related outcomes, such as post-operative complications, poor overall survival (OS) rate, disease progression-free survival rate, and extended hospitalization [16]. Notably, pharmacological interventions are unavailable for the prevention of developing sarcopenia and thereby impede its negative health outcomes and control its progression. Thus, the most effective approaches for its management rely on the strategies following lifestyle behavior modifications, including nutritional interventions [17]. Therefore, targeted nutritional interventions, including food supplements, are warranted to overcome muscle mass decline on time and ultimately control sarcopenia.

Nutritional interventions include adequate protein intake (leucine-enriched balanced creatine and amino acids) [18], antioxidant nutrients, vitamin D, long-chain polyunsaturated fatty acids [19], and beta-hydroxy-beta-methylbutyrate can help to reduce the developing risk of sarcopenia [20]. Likewise, vitamin C is another important antioxidant, and its deficiency, particularly in older females, can cause a higher risk of low muscle strength [21]. Similarly, vitamin E deficiency has been associated with low knee and grip strength [22]. In addition, lutein and zeaxanthin, magnesium, selenium, and omega-3 fatty acids can be used as supplements and demonstrate an association with muscle performance in older individuals [23,24]. Gut microbiota significantly and positively mediate the association between aging and nutrition by regulating the immune system, insulin activity, metabolism, and gene expression [25,26]. Numerous studies have investigated the impact of nutritional supplements on sarcopenia, but inconsistent outcomes have been reported. For instance, a branched chain of amino acids demonstrated effectiveness against different parameters associated with sarcopenia, like skeletal muscle index and muscle mass; however, non-significant improvement was observed in terms of handgrip strength [27]. In contrast, another study demonstrated improvement in the patient’s body weight, but no significant improvement was observed in the parameters associated with sarcopenia [28]. Similarly, low protein intake is associated with low muscle mass and strength across all ages.

To the best of our knowledge, no published systematic review and meta-analysis have described the effect of the combination of nutritional supplements on the progression of sarcopenia. Therefore, the present review was conducted to assess the impact of various nutrients on the progression and management of sarcopenia-associated problems, such as muscle mass, strength, and function. This review has great significance as it systematically synthesizes current evidence to clarify the role of specific nutrients in the prevention and management of sarcopenia, offering insights into dietary strategies that support musculoskeletal health in the aging population.

## Review

Methodology

Study Design

This review was conducted according to the 27-item guidelines of the Preferred Reporting Items for Systematic Reviews and Meta-analysis (PRISMA) to ensure the transparency and reproducibility of the outcomes [29].

Search Strategy

We engaged in a search for relevant literature on various databases, including PubMed, Scopus, ScienceDirect, The Cochrane Library, and Google Scholar, from January 2005 to May 2025. Different keywords and search terms were used, such as pathophysiology, disease mechanism, disease progression, underlying mechanisms, pathogenesis, nutrients, diet supplements, food supplement, proteins, creatine monohydrate, vitamins, vitamin D, vitamin D3, vitamin C, vitamin E, vitamin B1, vitamin B2, vitamin B6, vitamin B12, omega, magnesium, zinc, lycopene, lutein, zeaxanthins, antioxidants, sarcopenia, muscle wasting, muscle loss, skeletal muscle loss, muscular atrophy, and muscle decline. The Boolean operators (AND, OR) were used to combine the search terms for different databases, and the detailed search strategy is described in the table in the Appendices.

Inclusion Criteria

The inclusion criteria for selecting studies for intervention were based on the PICO guidelines: P (Population) - Patients aged >18 years from all settings. I (Interventions): Nutritional interventions. C (Control/Comparator): A well-defined control group with an alternative diet, placebo, standard care, or without any intervention. O (Outcomes): primary outcomes included changes in muscle mass, strength, skeletal mass area, handgrip, physical performance, and others. Secondary outcomes included biochemical markers (like inflammatory cytokines, etc.) relevant to sarcopenia. In addition, original interventional studies (RCTs, observational studies, clinical trials, cohort studies, case-control studies) published in peer-reviewed English journals were included.

Exclusion Criteria

The exclusion criteria were as follows: studies with insufficient data or those without control groups; studies that investigate non-nutritional interventions other than sarcopenia disease or other musculoskeletal diseases; and studies involving patients with cognitive or mental illness. Furthermore, animal studies, non-original studies, such as reviews (narrative scoping, systematic, meta-analysis), editorials, commentaries, letters, abstracts, and proceeding abstracts, were also excluded, as well as studies published in non-peer-reviewed journals and those in non-English languages.

Study Selection Process

**Figure 1:**
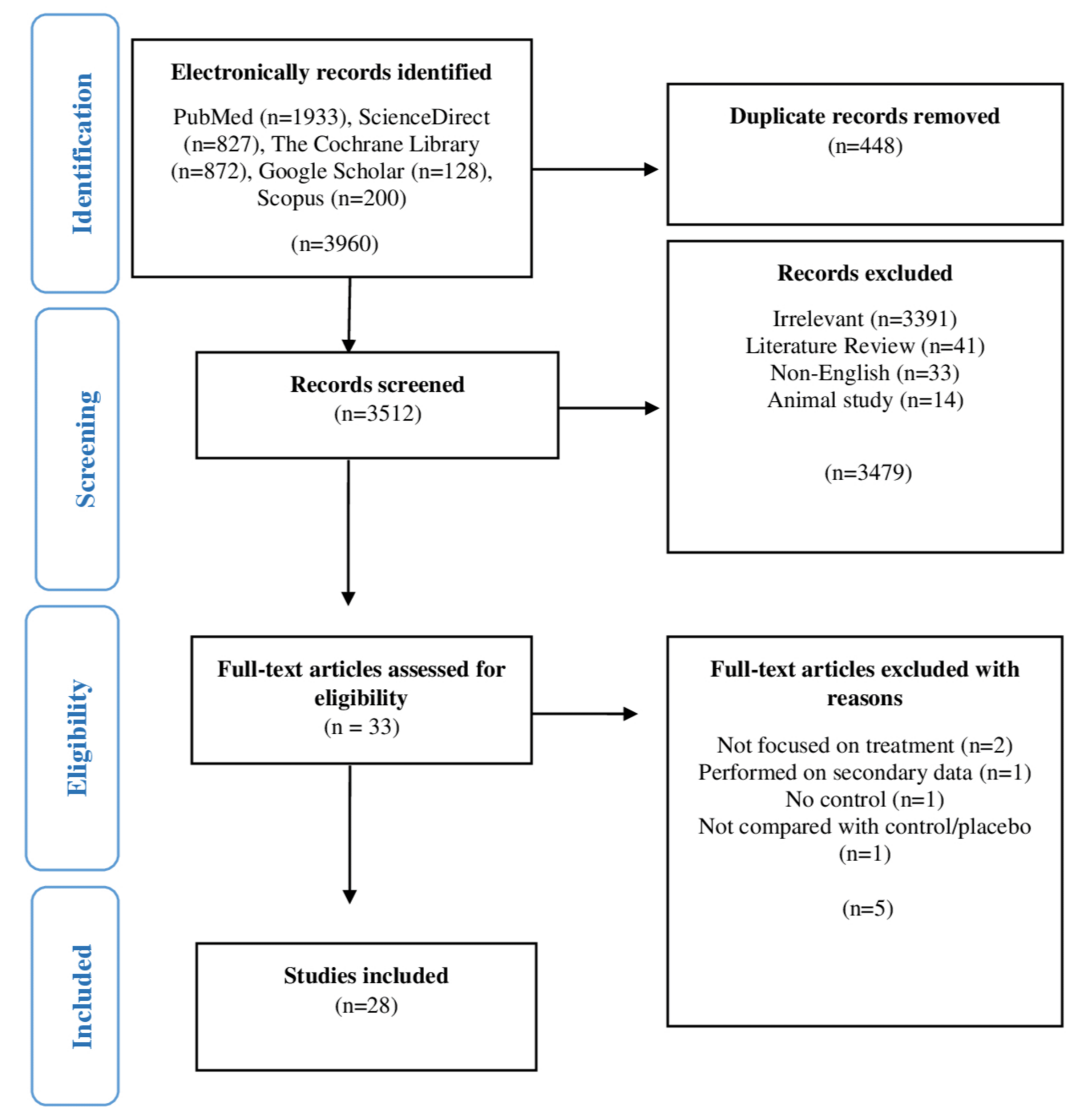
PRISMA flow chart depicting the selection of studies PRISMA: Preferred Items for Reporting Systematic Review and Meta-analysis

A four-stage process was employed for the selection of studies, as shown in the PRISMA flow chart in Figure 1. In the first stage (identification), 3960 studies were identified from different databases, moved to EndNote X9 referencing software, and 448 duplicate studies were removed. In the second stage (screening), 3512 studies were screened based on their titles and abstracts, and those deemed relevant to our study were advanced to the next stage, while 3479 non-relevant studies were excluded. In the third stage (eligibility), full-text assessment was performed on the remaining 33 studies following the inclusion/exclusion criteria. Twenty-eight studies were moved to the last stage of the selection process, and the remaining five studies were excluded for reasons explained in the PRISMA flow chart (Figure 1). In the last stage, 28 studies that fulfilled the criteria were included for further qualitative and quantitative analysis. This whole process was performed by two independent reviewers, and any discrepancy between the two reviewers was resolved by consulting a senior reviewer.

Data Extraction

Two independent reviewers extracted the data using a predefined data extraction form with different variables: study characteristics: author ID, study performed, country, study design, sample size; participant characteristics: gender, age, BMI; intervention characteristics: type of nutrition, composition, supplement dosage, exposure period, type of control, and physical exercise; and Outcomes: outcomes measured, adverse events, key outcomes, conclusion.

Methodological Quality Assessment

**Figure 2:**
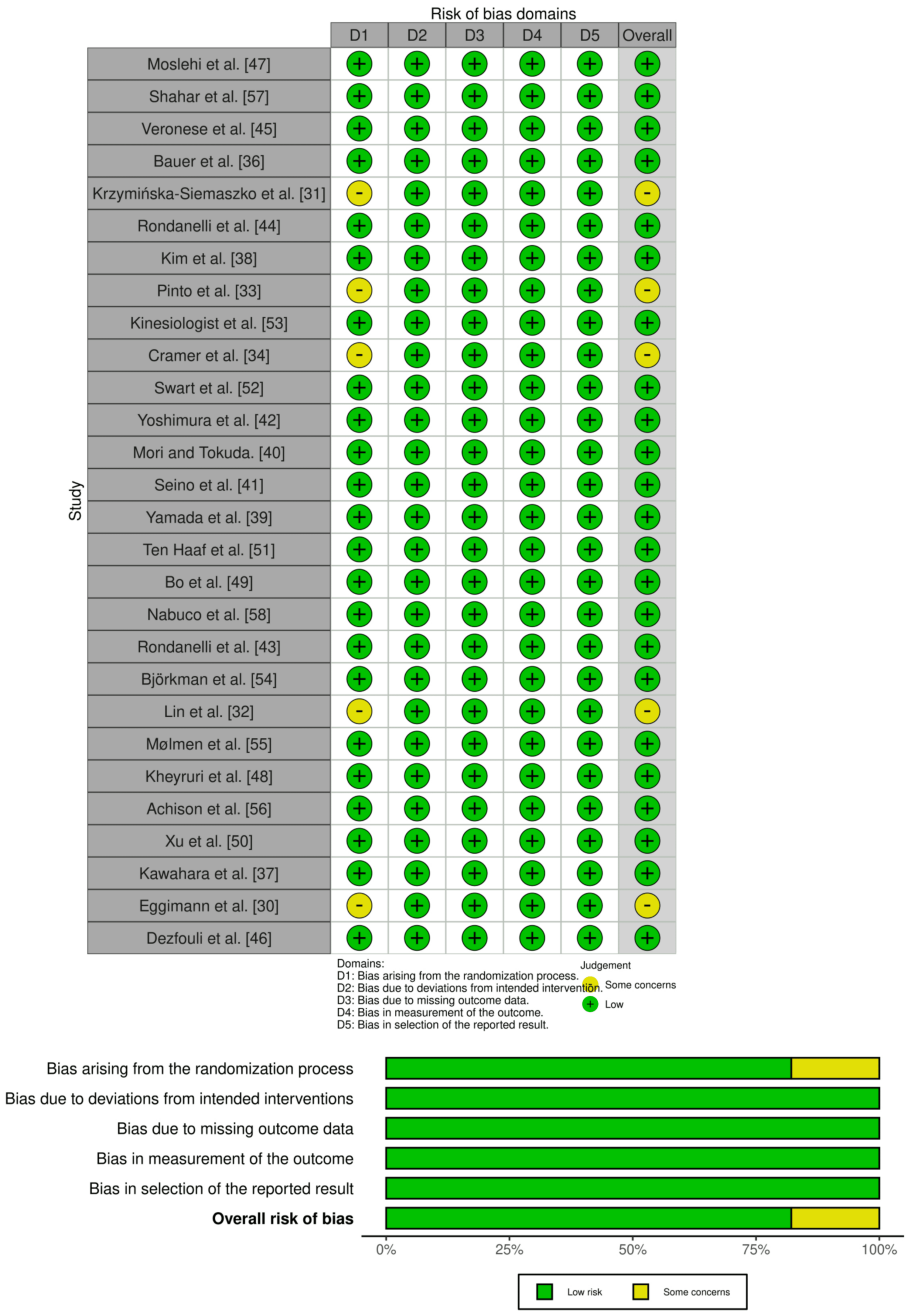
Methodological quality assessment of RCTs RCTs: randomized controlled trials

The Cochrane Risk of Bias-2.0 (RoB 2) was used for RCTs. Twenty-eight studies were characterized in each domain (randomization process, deviation from intended intervention, missing outcome data, outcome measurement, and selection of reported results) as either low, high, or having some concerns. All of the studies were found with low RoB, except for five studies, which had some concerns in the domain of the randomization process, as illustrated in Figure 2 [30-34]. Outcomes were reported in the form of visualization judgments associated with each RoB item and presented as percentages, and visualization of the assessed outcomes was performed using RobVis, a web-based tool [35]. The methodological quality assessment was performed by two independent reviewers.

Meta-Analysis

Qualitative data were presented in the table form, and key characteristics of studies and patients were summarized. Quantitative data were analyzed for the construction of forest plots using RevMan 5.4, and the association was measured using a chi-square test at the significance level of <0.05. Meanwhile, heterogeneity was calculated using I^2^ statistics, and heterogeneity of <25% (low), 26-75% (moderate), and >75% (high) was used. Publication bias was calculated using a funnel plot, and the distribution of studies that were symmetrical and indicated a clear funnel shape was deemed to indicate a low publication bias; in case of asymmetrical distribution of studies, a clear funnel shape was not formed, indicating higher publication bias.

Certainty of Evidence

The Grading, Reporting, Assessment, Development, and Evaluation (GRADE) framework was used for the assessment of the certainty of the evidence of the outcomes. Two independent reviewers categorized the outcomes as low, moderate, or high certainty of evidence in the context of precision, indirectness, publication bias, risk of bias, and any other risk.

Results

General Characteristics of the Included Studies

All studies were published during 2013-2025, and most of them were single-centered, except for three studies, which were multicentered [30,34,36]. Most of the single-centered studies were reported from Japan [37-42], followed by Italy [43-45], Iran [46-48], China [49,50], and the Netherlands [51,52]. The single-center studies were reported from Chile [53], Brazil [33], Finland [54], Norway [55], Poland [31], the UK [56], Malaysia [57], Brazil [58], and Taiwan [32]. All studies were RCTs, as outlined in Table 1. A varied sample size was utilized for performing these trials; the minimum and maximum samples were 13 for the intervention group and 14 for the control group [33], and 1461 for the intervention group and 1458 for the control group, respectively [52]. In terms of age, the age of the elderly patients ranged from 60.7 to 84.9 years for the intervention group [37,39]; for the control group, this range was 60.9 to 84.7 years [37,39]. Most of the studies were skewed towards the inclusion of females, and two studies included 100% females for intervention and control [38,53]. The BMI of the study participants also varied and ranged from 19.74 kg/m^2^ to 29.5 kg/m^2^, as detailed in Table 1.

**Table 1:** Summary of the general characteristics of the included studies and participants BMI: body mass index; NA: not available; RCT: randomized controlled trial

Study ID	Study performed	Country/region	Study design	Sample size	Age, years	Gender (M/F)	BMI
Moslehi et al., 2013 [47]	Single center	Iran	RCT	Intervention group=35, control group=34	Intervention group=46.5, control group=46.1	NA	Intervention group=28, control group=28.1
Shahar et al., 2013 [57]	Single center	Malaysia	RCT	Intervention group=15, control group=15	Intervention group=65.93, control group=67.25	NA	Intervention group=24.26, control group=26.36
Veronese et al., 2014 [45]	Single center	Italy	RCT	Intervention group=53, control group=71	71.5	NA	NA
Bauer et al., 2015 [36]	Multicenter	Germany, Belgium, Ireland, Sweden, Italy, UK	RCT	Intervention group=184, control group=196	Intervention group=77.3, control group=78.1	Intervention group=64:120, control group=66:129	Intervention group=26, control group=26.2
Krzymińska-Siemaszko et al., 2015 [31]	Single center	Poland	RCT	Intervention group=30, control group=20	Intervention group=74.97, control group=74.85	Intervention group=11:19, control group=6:14	Intervention group=23.41, control group=22.93
Rondanelli et al., 2016 [44]	Single center	Italy	RCT	Intervention group=69, control group=61	Intervention group=80.77, control group=80.21	Intervention group=29:40, control group=24:37	Intervention group=23.85, control group=23.93
Kim et al., 2016 [38]	Single center	Japan	RCT	Intervention group 1 (nutrition+exercise)=36, control group 1 (exercise alone)=35; intervention group 2 (nutrition alone)=34, control group 2 (health education)=34	Intervention group 1 (nutrition + exercise)=80.9, control group 1 (exercise alone)=81.4; intervention group 2 (nutrition alone)=81.2, control group 2 (health education)=81.1	Females: 100%	Intervention group 1 (nutrition+exercise)=24.9, control group 1 (exercise alone)=25.1; intervention group 2 (nutrition alone)=24.9, control group 2 (health education)=25.1
Pinto et al., 2016 [33]	Single center	Brazil	RCT	Intervention group=13, control group=14	Intervention group=67.4, control group=67.1	NA	NA
Kinesiologist et al., 2016 [53]	Single center	Chile	RCT	Intervention group=23, control group=25	Intervention group=67.5, control group=68.1	Females: 100%	Intervention group=29.2, control group=29.5
Cramer et al., 2016 [34]	Multicenter	Europe and North America: 8 countries	RCT	Intervention group=165, control group=165	Intervention group=77, control group=77	Intervention group=38%: 62%, control group=38%: 62%	Intervention group=25, control group=26
Swart et al., 2016 [52]	Single center	The Netherlands	RCT	Intervention group=1461, control group=1458	Intervention group=74.2, control group=74	Intervention group=49.7% F, control group=50.1% F	NA
Yoshimura et al., 2016 [42]	Single center	Japan	RCT	Intervention group=19, control group=17	Intervention group=78.4, control group=82.3	Intervention group=4:15, control group=6:11	Intervention group=20.8, control group=20.4
Mori and Tokuda, 2018 [40]	Single center	Japan	RCT	Intervention group 1=25, intervention group 2=25, control group=25	Intervention group 1=70.6, intervention group 2=70.6, control group=70.6	NA	Intervention group 1=22.3, intervention group 2=22.1, control group=22.9
Seino et al., 2018 [41]	Single center	Japan	RCT	Intervention group=40, control group=40	Intervention group=73.4, control group=73.7	Intervention group=6:34, control group=7:33	Intervention group=22.9, control group=22.9
Yamada et al., 2019 [39]	Single center	Japan	RCT	Intervention group 1 (nutrition + exercise)=28, control group 1 (exercise alone)=28; intervention group 2 (nutrition alone)=28, control group 2 (health education)=28	Intervention group 1 (nutrition + exercise)=84.9, control group 1 (exercise alone)=84.7; intervention group 2 (nutrition alone)=83.2, control group 2 (health education)=83.9	Intervention group 1 (nutrition + exercise)=8:20, control group 1 (exercise alone)=10:18; intervention group 2 (nutrition alone)=8:20, control group 2 (health education)=13:15	Intervention group 1 (nutrition+exercise)=21.3, control group 1 (exercise alone)=22.6; intervention group 2 (nutrition alone)=22.6, control group 2 (health education)=21.2
Ten Haaf et al., 2019 [51]	Single center	The Netherlands	RCT	Intervention group=58, control group=56	Intervention group=69, control group=69	Intervention group=47:11, control group=46:10	Intervention group=27.2, control group=26.3
Bo et al., 2019 [49]	Single center	China	RCT	Intervention group=30, control group=30	Intervention group=73.23, control group=74.83	Intervention group=13:17, control group=14:16	Intervention group=21.34, control group=19.74
Nabuco et al., 2019 [58]	Single center	Brazil	RCT	Intervention group=13, control group=13	Intervention group=68, control group=70.1	NA	Intervention group=26.4, control group=27.4
Rondanelli et al., 2020 [43]	Single center	Italy	RCT	Intervention group=70, control group=70	Intervention group=80, control group=81	Intervention group=29:41, control group=23:47	Intervention group=21.1, control group=22.1
Björkman et al., 2020 [54]	Single center	Finland	RCT	Intervention group=73, placebo group=73, no supplementation or placebo group=72	Intervention group=83.6, placebo group=84, no supplementation or placebo group=83.7	Intervention group=69.9% F, placebo group=62.5% F, no supplementation or placebo group=70.8% F	Intervention group=25.3, placebo group=26.8, no supplementation or placebo group=26.3
Lin et al., 2021 [32]	Single center	Taiwan	RCT	Intervention group=28, control group=28	Intervention group=73.8, control group=72.5	Intervention group=19:9, control group=21:7	Intervention group=19.8, control group=20.6
Mølmen et al., 2021 [55]	Single center	Norway	RCT	Intervention group=46, control group=48	Intervention group=69, control group=67	Intervention group=22:24, control group=21:27	Intervention group=26, control group=26
Kheyruri et al., 2021 [48]	Single center	Iran	RCT	Intervention group=42, control group=41	Intervention group=45, control group=47	NA	Intervention group=32.35, control group=31
Achison et al., 2022 [56]	Single center	UK	RCT	Intervention group=72, control group=73	Intervention group=78.3, control group=79.3	Intervention group=34:38, control group=33:40	Intervention group=27.1, control group=26.5
Xu et al., 2022 [50]	Single center	China	RCT	Intervention group=100, control group=100	Intervention group=66.63, control group=67.31	Intervention group=42:58, control group=42:58	Intervention group=25.64, control group=24.63
Kawahara et al., 2024 [37]	Single center	Japan	RCT	Intervention group=548, control group=546	Intervention group=60.7, control group=60.9	Intervention group=303:245, control group=307:239	Intervention group=24.5, control group=24.5
Eggimann et al., 2025 [30]	Multicenter	Austria, France, Germany, Portugal, and Switzerland	RCT	Intervention group 1 (vitamin D)=746, control group 1=749; intervention group 2 (Omega 3)=752, control group 2=743	Intervention group 1 (vitamin D)=75, control group 1=74.9; intervention group 2 (Omega 3)=74.9, control group 2=75	Intervention group 1 (vitamin D)=273:476, control group 1=279:470; intervention group 2 (omega-3)=279:473, control group 2=270:473	Intervention group 1 (vitamin D)=26.8, control group 1=26.5; intervention group 2 (omega- 3)=26.8, control group 2=26.7
Dezfouli et al., 2025 [46]	Single center	Iran	RCT	Intervention group=24, control group=24	Intervention group=69.33, control group=69.20	Intervention group=16:8, control group=14:10	Intervention group=22.66, control group=21.81

Characteristics of Intervention

Most of the interventions aimed at improving health outcomes, often in conjunction with physical exercise; however, there were studies that combined nutritional intervention with physical exercise to enhance the clinical outcomes of the patients [33,40-42,44,57,58]. Nutritional interventions include protein supplements [32,43,51,54], omega-3 [30,50], vitamin D [30,37,55], creatine supplements [33,53], amino acids [38], magnesium [45,47], vitamin B12 [52], and leucine [56]. Most of the studies used a combination of nutrients, including zinc and other micronutrients, for the enhancement of muscle mass, strength, and other physical functional outcomes [31,36,39,44,46,49]. Dosages and duration vary from daily intakes of 1.2-40 g [32,36] over periods ranging from four weeks to three years [30,37,43]. For comparison as a control, most studies used a placebo [37,39,44,46,50,51,53,55], iso-caloric as a control product [36,43,49,54], and other materials, as presented in Table 2.

**Table 2:** Summary of the characteristics of nutritional interventions DHA: docosahexaenoic acid; EPA: eicosapentaenoic; HCL: hydrochloric acid; NA: not available

Study ID	Type of intervention	Composition	Dose	Duration	Control	Physical exercise
Moslehi et al., 2013 [47]	Magnesium supplement	Magnesium=250 mg	One table/daily	8 weeks	Placebo	No
Shahar et al., 2013 [57]	Protein supplement	Protein=23%, fat=0.8%, carbohydrates=0.3%	1.5 g/kg/day	12 weeks	Placebo	Yes (resistance training)
Veronese et al., 2014 [45]	Magnesium supplement	Magnesium	300 mg/day	12 weeks	Placebo	Yes
Bauer et al., 2015 [36]	Vitamin D and leucine-enriched whey protein	Whey protein=20 g, carbohydrate=9 g, total leucine=3 g, fat=3 g, vitamin D=800 IU; a mixture of vitamins, minerals, and fibers	40g/ 100-150 mL water	13 weeks	Iso-caloric	No
Krzymińska-Siemaszko et al., 2015 [31]	Omega-3 + vitamin E	EPA=660 mg, DHA=440 mg, omega-3 fatty acids=200mg, vitamin E=10 mg	1.3 g	12 weeks	Vitamin E	No
Rondanelli et al., 2016 [44]	Protein, amino acids, and vitamin D + physical activity	Protein=22 g, essential amino acids=10.9 g, (leucine [4 g]), vitamin D=2.5 mg (100 IU)	32 g	12 weeks	Placebo	Yes (strengthening exercise)
Kim et al., 2016 [38]	Amino acid supplement	Leucine-enriched essential amino acids (leucine [1.20 g], lysine HCl [0.5 g], valine [0.33 g], isoleucine [0.32 g], threonine [0.28 g], phenylalanine 0.20 g, others [0.17 g]=3 g, vitamin D=20 mg	NA	3 months	Control=exercise, control 2=health education	Yes (aerobic training, resistance, and weight-bearing exercise)
Pinto et al., 2016 [33]	Creatine supplement + resistance training	Creatine monohydrate	5 g/day	12 weeks	Placebo + resistance training	Yes (resistance training)
Kinesiologist et al., 2016 [53]	Creatine supplement	Creatine	5 g	3 months	Placebo	Yes (resistance training)
Cramer et al., 2016 [34]	High protein supplement	Protein=20 g, fat=11 g, carbohydrate=36 g, vitamin D3=12 µg, vitamin B12=13 µg, magnesium=55 mg, zinc=3.9 mg, other minerals and vitamins	220 mL/twice daily	24 weeks	Iso-caloric	No
Swart et al., 2016 [52]	Vitamin B12 and folic acid	Vitamin B12=500 µg, folic acid=400 µg, vitamin D3=600 IU	Daily	2 years	Placebo + vitamin D3	No
Yoshimura et al., 2016 [42]	Nutritional supplement + exercise	Protein=10 g, amino acids=2500 mg, fat=8.2 g, carbohydrate=20.6 g, vitamin D=12.5 µg, vitamin B12=125 µg, others	Daily	2-6 months	Exercise	Yes (resistance training)
Mori and Tokuda, 2018 [40]	Whey protein supplement	Energy=92 kcal, protein=23 g, fat=0.3 g, carbohydrate=0.1 g, valine=1225 mg, leucine=2975 mg	1.2 g/kg/day	24 weeks	Exercise only	Yes (resistance training)
Seino et al., 2018 [41]	Dairy protein + micronutrients + exercise	Dairy protein=10.5 g, zinc=0.8 mg, vitamin B12=12 µg, folic acid=200 µg, vitamin D=200 IU	Twice/daily	12 weeks	Exercise	Yes (resistance training)
Yamada et al., 2019 [39]	Protein supplement + vitamin D	Energy=100 kcal, whey protein=10 g, vitamin D=800 IU	NA	12 weeks	Placebo	Yes (resistance training)
Ten Haaf et al., 2019 [51]	Protein supplement	Protein=31 g, fat=1.1 g, carbohydrate=14.5 g	36.8 g	12 week	Placebo	Yes (walking exercise)
Bo et al., 2019 [49]	Whey protein, vitamin D, and E supplement	Protein=22 g, carbohydrate=10.4 g, fats=2.6 g, vitamin D=702 IU, vitamin E=109 mg	40 g powder to be reconstituted with 100-150 mL water per serving	6 months	Iso-caloric	No
Nabuco et al., 2019 [58]	Whey protein supplement	Protein=35 g	Daily	12 weeks	Placebo + exercise	Yes (resistance training)
Rondanelli et al., 2020 [43]	Protein supplement	Whey protein=20 g, leucine=2.8 g, carbohydrates=9 g, fat=3 g, vitamin D and minerals	34.8 g/ twice daily	4-8 weeks	Iso-caloric	Yes (physical fitness and muscle mass promoting program)
Björkman et al., 2020 [54]	Protein supplement	Whey protein=20 g, vitamin D=20 µg	Twice/daily	12 months	Iso-caloric and no supplement group	Yes (home-based exercise)
Lin et al., 2021 [32]	Protein supplement	Energy=88 kcal, protein=12.8 g (including whey protein concentrate [8.5 g]), leucine=1.2 g, carbohydrates=7.3 g, fat=0.8 g, vitamin D=120 IU	1.2-1.5 g/kg/day in 200 mL water	12 weeks	Ordinary protein-rich diet via counseling	No
Mølmen et al., 2021 [55]	Vitamin D3 supplement	Vitamin D3	Initially for 2 weeks=10 000 IU/day, remaining period=2000 IU/day	2 months	Placebo	Yes (resistance training)
Kheyruri et al., 2021 [48]	Vitamin D and magnesium co-supplement	Vitamin D=50 IU, magnesium=250 mg	Vitamin D=weekly, magnesium=daily	8 weeks	Placebo	No
Achison et al., 2022 [56]	Leucine powder	Leucine	2.5 g/thrice a day	12 months	No leucine	No
Xu et al., 2022 [50]	Fish oil-derived n-3 polyunsaturated fatty acid	EPA=1.34 g, DHA=1.07 g	4 g/day	6 months	Placebo	No
Kawahara et al., 2024 [37]	Eldecalcitol	Active vitamin D	0·75 μg per day	3 years	Placebo	No
Eggimann et al., 2025 [30]	Intervention 1=vitamin D, ntervention 2=omega-3 supplementation	Vitamin D and marine omega-3	Vitamin D=2000 IU/day, marine omega-3=1 g/day	3 years	No supplements	Yes (home-based exercise)
Dezfouli et al., 2025 [46]	Sarcomeal® oral supplementation plus vitamin D3	Sarcomeal (whey protein, creatine, branched chain amino acids, glutamine) + vitamin D3	Sarcomeal=38 g/day and vitamin D3=1000 IU	12 weeks	Placebo	Yes (resistance training)

Outcomes

Numerous interventions, like protein, amino acids, vitamin D, creatine, magnesium, vitamin B12, and zinc or other minerals, have frequently been demonstrated to produce significant and enhanced improvements in muscle mass, strength, and physical function [33,43-45,57]. Likewise, nutritional interventions are also reported to be beneficial in improving body composition, strength, and gait speed [32,37,50,57]. However, non-significant improvement was also observed in the intervention group, when compared with the control group [30,31,53,56], indicating variability in response or potential limitations in the study population, intervention protocols, type, dose, and formulations. Meanwhile, nutritional intervention, when combined with physical exercise, did improve the outcomes associated with muscle mass and physical performance [33,38-42,44,58]. These findings further support the value of targeted nutritional strategies in the aging population. Adverse events were generally not serious, as indicated in Table 3. These findings are consistent with a broader body of evidence suggesting that nutritional supplements play a very important role in supporting the health of individuals with or at risk of sarcopenia or any other functional decline.

**Table 3:** Summary of outcomes GI: gastrointestinal; hs-CRP: high sensitivity C-reactive protein; IL: interleukin; NA: not available; SPPB: short physical performance battery

Study ID	Outcome measured	Adverse events	Key findings	Conclusion
Moslehi et al., 2013 [47]	Body composition, muscle strength	NA	Non-significant difference was observed between the groups	Intervention did not significantly improve the body composition and strength
Shahar et al., 2013 [57]	Body composition, physical function, oxidative stress	NA	Significantly reduced body weight and improved strength	Intervention improves the strength
Veronese et al., 2014 [45]	Physical performance	No serious adverse events	Significantly improved	Intervention improve the physical performance
Bauer et al., 2015 [36]	Handgrip strength, SPPB score, chair-stand test, gait speed, balance score, and appendicular muscle mass	Non-significant differences in the incidence of serious adverse events	Handgrip strength and SPPB: non-significant improvement in both groups; appendicular muscle mass and chair-stand test: the intervention group demonstrated significant improvement	Intervention resulted in improvements
Krzymińska-Siemaszko et al., 2015 [31]	Body composition, muscle strength, and physical performance	Gastro-intestinal problems	Average muscle strength: non-significant difference; walking test (4 meter) and timed up and go test: pre-post difference in both groups	No impact on the study variable after treatment
Rondanelli et al., 2016 [44]	Fat-free mass, strength, functionality, quality of life, reduction in inflammation	No serious adverse events	Fat-free mass, relative skeletal muscle mass, android distribution of fat, and handgrip strength significantly increased in the intervention group	Effective when combined with physical exercise
Kim et al., 2016 [38]	Body composition, physical function (grip strength), biomarkers (IL-6, hs-CRP)	No serious adverse events	Total body fats: significantly decreased in the intervention group and improved walking speed; biomarkers: non-significant impact	The combination of exercise and nutrition effectively improved the study variables except for biomarkers
Pinto et al., 2016 [33]	Lean mass	No serious adverse events	Significantly higher gain of lean mass in the intervention group	Supplement in combination with resistance training becomes effective
Kinesiologist et al., 2016 [53]	Muscle mass and function	In the treatment group, one patient reported a gastric ulcer	Non-significant difference in the outcomes	Intervention did not demonstrate effectiveness
Cramer et al., 2016 [34]	Gait speed, strength	Gastrointestinal	Non-significant improvement in gait speed and strength	Both interventions had comparable outcomes
Swart et al., 2016 [52]	Physical performance, strength, falling	NA	Non-significant impact on the study variables except for gait speed	Outcomes should be further validated
Yoshimura et al., 2016 [42]	Body composition, physical function	NA	Significant improvement was observed	Intervention combined with exercise improves the study variables
Mori and Tokuda, 2018 [40]	Gait speed, strength	NA	Significantly increased handgrip strength and gait speed in the exercise + supplement group than the supplement alone and control group	Supplement combined with resistance exercise improves the study outcomes
Seino et al., 2018 [41]	Muscle mass, gait speed	No serious adverse events	Significant improvement in muscle mass and non-significant improvement in gait speed in the intervention group	Supplement when combined with exercise, it increases muscle mass but no impact on physical function
Yamada et al., 2019 [39]	Skeletal muscle mass	No serious adverse events	Appendicular muscle mass: significantly increased in the intervention group	Combined nutrition and exercise had great impact
Ten Haaf et al., 2019 [51]	Body composition, strength, physical performance	No serious adverse events	Lean body mass: a significantly larger increase in the intervention group; strength and contractile function did not significantly change in both groups	Intervention when combined with exercise becomes more effective
Bo et al., 2019 [49]	Muscle mass, strength, biomarkers	No serious adverse events	Muscle mass, relative skeletal mass index, strength, and biomarkers (IL-2): significantly improved	Intervention effectively improved the study variables
Nabuco et al., 2019 [58]	Body composition, physical function, and inflammation biomarker	NA	Significantly increased trunk mass, lean mass, and inflammation biomarkers	Supplement combined with exercise improve the clinical outcomes
Rondanelli et al., 2020 [43]	Gait speed, muscle strength, and physical performance	No serious adverse events	Gait speed, muscle mass, and functional performance: significantly improved in the intervention group	Intervention effectively improved functional and physical performance
Björkman et al., 2020 [54]	Physical performance, hand grip strength, calf intracellular resistance	56% adverse events occurred in the supplemented and placebo group, while 9% reported in the control group	Physical performance and calf intracellular resistance-based skeletal muscle index: non-significant difference; hand grip strength: significant difference between both groups	Supplement combined with low intensity home-based physical exercise did not attenuate the deterioration of muscle and physical performance
Lin et al., 2021 [32]	Muscle mass, handgrip strength, gait speed	NA	Appendicular muscle mass index and handgrip strength: non-significant difference; gait speed: significantly improved in the intervention group	Supplement improves the protein intake
Mølmen et al., 2021 [55]	Muscle mass, strength, and physical performance	No serious adverse events	Non-significant effect on training-associated changes for any of the main outcome domains	Intervention combined with exercise did not enhance the outcomes
Kheyruri et al., 2021 [48]	Muscle mass, strength, physical performance, and inflammation	NA	Significant impact on the handgrip strength and inflammation biomarkers, while non-significant impact on leg extension strength and body mass	Intervention had a beneficial impact
Achison et al., 2022 [56]	Physical performance, muscle mass	Death in the control group, and fracture in both groups	Physical performance and muscle mass: non-significant impact	Intervention did not improve the physical performance and muscle mass
Xu et al., 2022 [50]	Physical performance, body composition, and strength	No serious adverse events	Physical performance, body composition, and strength: significantly improved in the intervention group	Fish oil supplements had benefits
Kawahara et al., 2024 [37]	Skeletal muscle mass, strength	Serious adverse events (respirator, cardio, GI, skin, urogenital, muscle skeletal system) occurred in both groups	Significant reduction in falls, grip strength, skeletal mass index, and fat mass index	Intervention has the potential to prevent the onset of sarcopenia via increasing skeletal muscle volume and strength
Eggimann et al., 2025 [30]	Appendicular lean muscle mass	NA	Appendicular lean muscle mass: non-significant difference compared to control over 3 years; however, omega-3 showed a small protective effect at year 1 only	No impact on the study variable after treatment
Dezfouli et al., 2025 [46]	Muscle parameters	No serious adverse events	Mean skeletal muscle mass index and grip strength: non-significant. However, significant difference in lean mass and lean mass index	The combination brings improvement in physical function and maintains weight

Meta-analysis

Handgrip Strength

**Figure 3:**
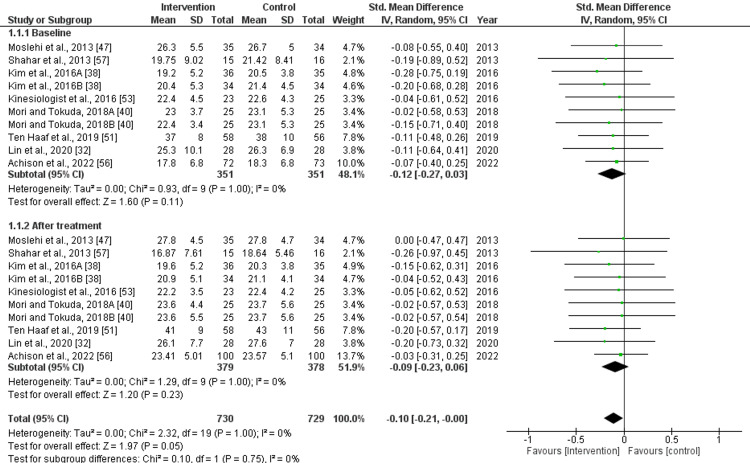
Forest plot for the assessment of handgrip strength before and after the intervention (nutritional supplementation) and in comparison with control/placebo Kim et al., 2016A indicates data for nutrition + exercise, and Kim et al., 2016B indicates nutrition alone. Similarly, Mori and Tokuda, 2018A indicates data for nutrition only, and Mori and Tokuda, 2018B indicates nutrition + exercise CI: confidence interval; SD: standard deviation

The baseline pooled effect size for handgrip strength demonstrated a std. mean difference (MD) of -0.12 (95% CI: -0.27 to 0.03), with a non-significant difference (p=0.11) and low heterogeneity (I^2^=0%). After treatment with nutritional supplements, the pooled effect size was as follows: std. MD: -0.09 (95% CI: -0.23 to 0.06), with non-significant (p=0.23) difference and low heterogeneity (I^2^=0%). However, the overall pooled effect size was as follows: std. MD: -0.10 (95% CI: -0.21 to 0.00), with a slightly significant (p=0.05) difference and low heterogeneity (I^2^=0%), as illustrated in Figure 3. This indicates that nutritional supplements had an impact on the handgrip strength. 

Skeletal Muscle Mass Index (Kg/m^2^) and Skeletal Muscle Mass (Kg)

**Figure 4:**
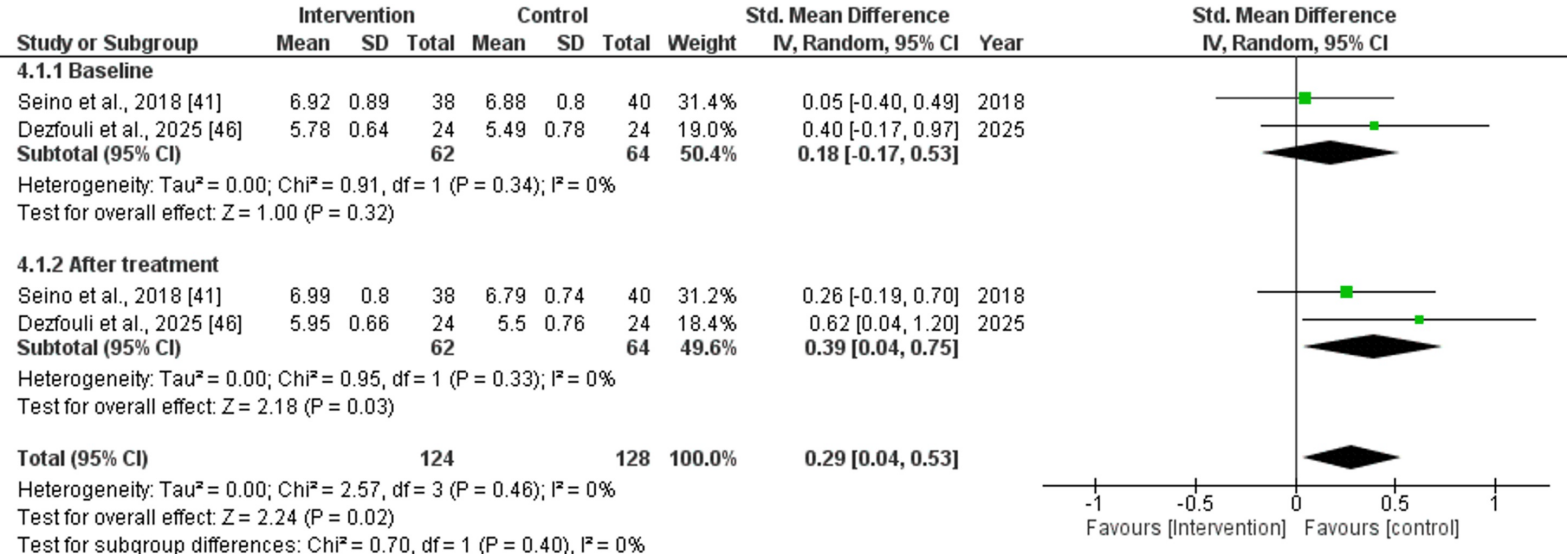
Forest plot for the assessment of skeletal muscle mass index (Kg/m2) before and after the intervention (nutritional supplementation) and in comparison with control/placebo CI: confidence interval; SD: standard deviation

**Figure 5:**
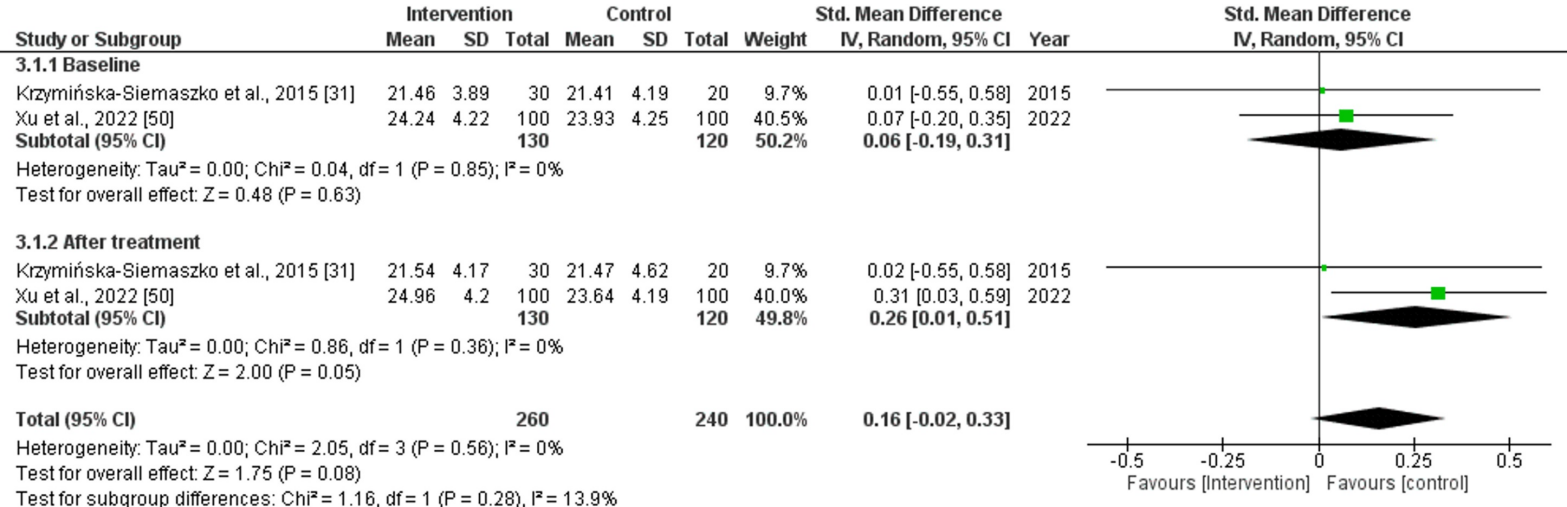
Forest plot for the assessment of skeletal muscle mass (Kg) before and after the intervention (nutritional supplementation) and in comparison with control/placebo CI: confidence interval; SD: standard deviation

The pooled effect size for skeletal muscle mass index and skeletal muscle mass before the treatment was as follows: std. MD: 0.18 (95% CI: -0.17 to 0.53), with a non-significant difference, and std. MD: 0.09 (95% CI: -0.19 to 0.36), with non-significant differences (p=0.32 and 0.85) and low heterogeneity (I^2^=0%). After treatment with nutritional supplements, the pooled effect size was as follows: std. MD: 0.39 (95% CI: 0.04 to 0.75), with significant difference (p=0.03) and std. MD: 0.26 (95% CI: 0.01 to 0.51), with a slightly significant (p=0.05) difference and low (I^2^=0%) heterogeneity. Overall, the pooled effect size was as follows: std. MD: 0.29 (95% CI: 0.04 to 0.53), with significant difference (p=0.02, I^2^=0%) for skeletal muscle mass index and std. MD: 0.16 (95% CI: -0.02 to 0.33, I2=0%), with a slightly non-significant (p=0.08) difference for skeletal muscle mass, as depicted in Figures 4, 5. 

Total Fat Mass

**Figure 6:**
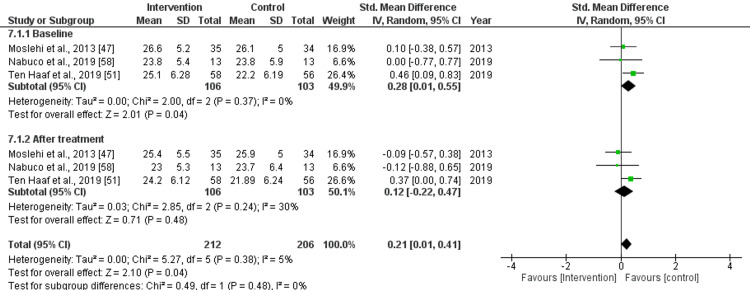
Forest plot for the assessment of total fat mass before and after the intervention (nutritional supplementation) and in comparison with control/placebo CI: confidence interval; SD: standard deviation

The pooled effect size for total fat mass before the treatment was as follows: std. MD: 0.28 (95% CI: 0.01 to 0.55), with significant differences (p=0.04) and low heterogeneity (I^2^=0%). After treatment with nutritional supplements, the pooled effect size was as follows: std. MD: 0.12 (95% CI: -0.22 to 0.47), with non-significant (p=0.48) difference and moderate heterogeneity (I^2^=30%). Overall, the pooled effect size was as follows: std. MD: 0.21 (95% CI: 0.01 to 0.41), with significant (p=0.04) differences and low heterogeneity (I^2^=5%), as shown in Figure 6.

Appendicular Lean Mass (Kg)

**Figure 7:**
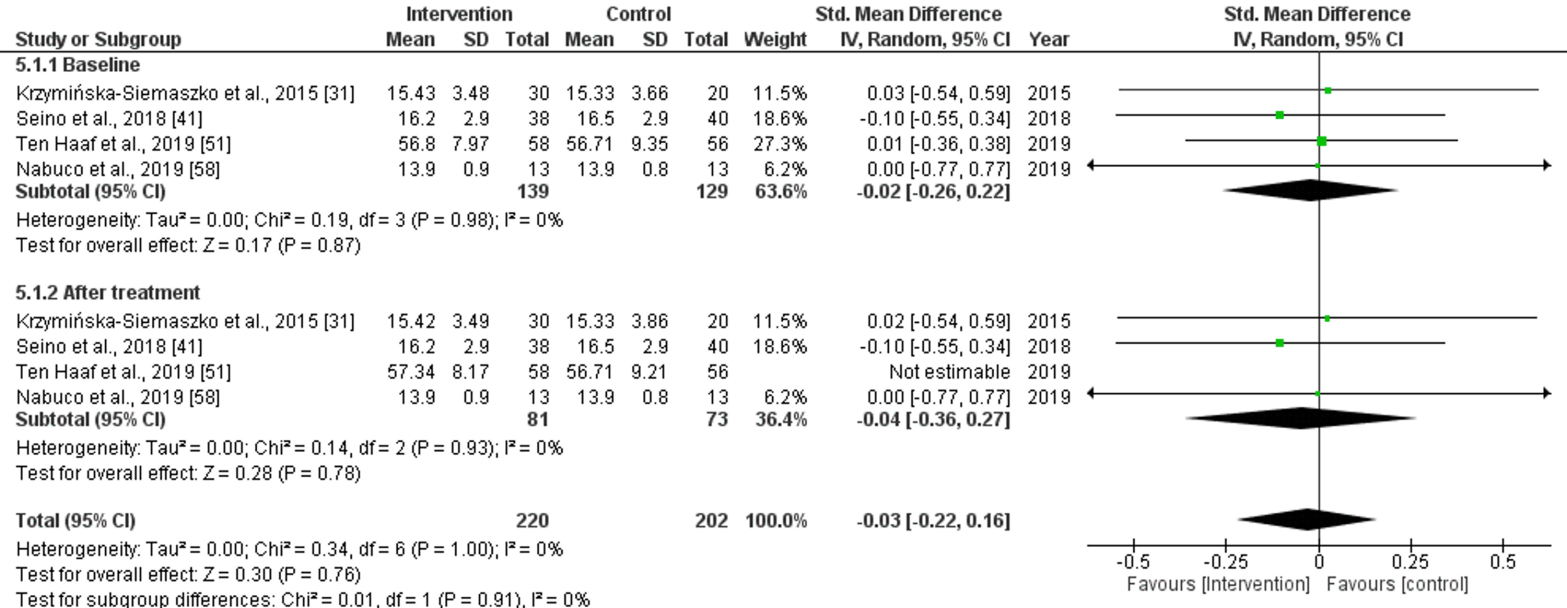
Forest plot for the assessment of appendicular lean mass (Kg) before and after the intervention (nutritional supplementation) and in comparison with control/placebo CI: confidence interval; SD: standard deviation

The baseline pooled effect size for appendicular lean mass (Kg) demonstrated a std. MD of -0.02 (95% CI: -0.26 to 0.22), with non-significant difference (p=0.87) and low heterogeneity (I^2^=0%). After treatment with nutritional supplements, the pooled effect size was as follows: std. MD: -0.04 (95% CI: -0.36 to 0.27), with non-significant (p=0.78) difference and low heterogeneity (I^2^=0%). Overall, the pooled effect size was as follows: std. MD: -0.03 (95% CI: -0.22 to 0.16), with non-significant (p=0.76) difference and low heterogeneity (I^2^=0%), as presented in Figure 7.

Gait Speed (m/s)

**Figure 8:**
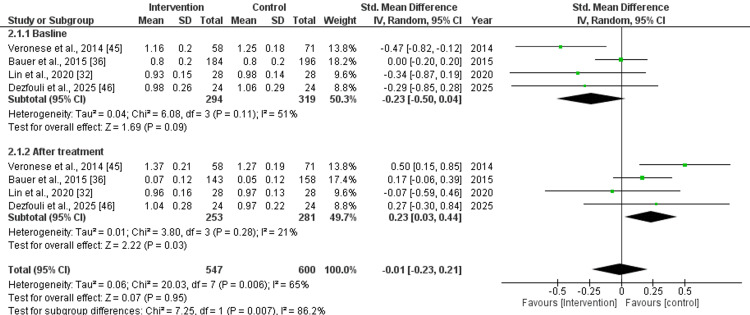
Forest plot for the assessment of gait speed (m/s) before and after the intervention (nutritional supplementation) and in comparison with control/placebo CI: confidence interval; SD: standard deviation

Before treatment, the baseline pooled effect size for gait speed was as follows: std. MD: -0.23 (95% CI: -0.50 to 0.04), with non-significant difference (p=0.09) and moderate heterogeneity (I^2^=51%). After treatment with nutritional supplements, the pooled effect size was as follows: std. MD: 0.23 (95% CI: 0.03 to 0.44), with significant (p=0.03) difference and low heterogeneity (I^2^=21%). Overall, the pooled effect size was as follows: std. MD: 0.01 (95% CI: -0.23 to 0.21), with non-significant (p=0.95) difference and moderate heterogeneity (I^2^=65%), as depicted in Figure 8.

Adverse Events

**Figure 9:**
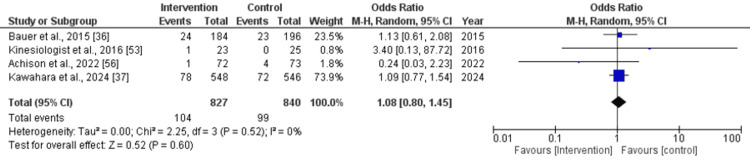
Forest plot for the serious adverse events that occurred after the intervention (nutritional supplementation) and in comparison with control/placebo CI: confidence interval

The pooled effect size for adverse events was as follows: odds ratio (OR): 1.08 (95% CI: 0.80-1.45), with a non-significant difference (p=0.60) between the intervention and control group and low heterogeneity (I^2^=0%) observed across the studies (Figure 9).

Publication Bias

**Figure 10:**
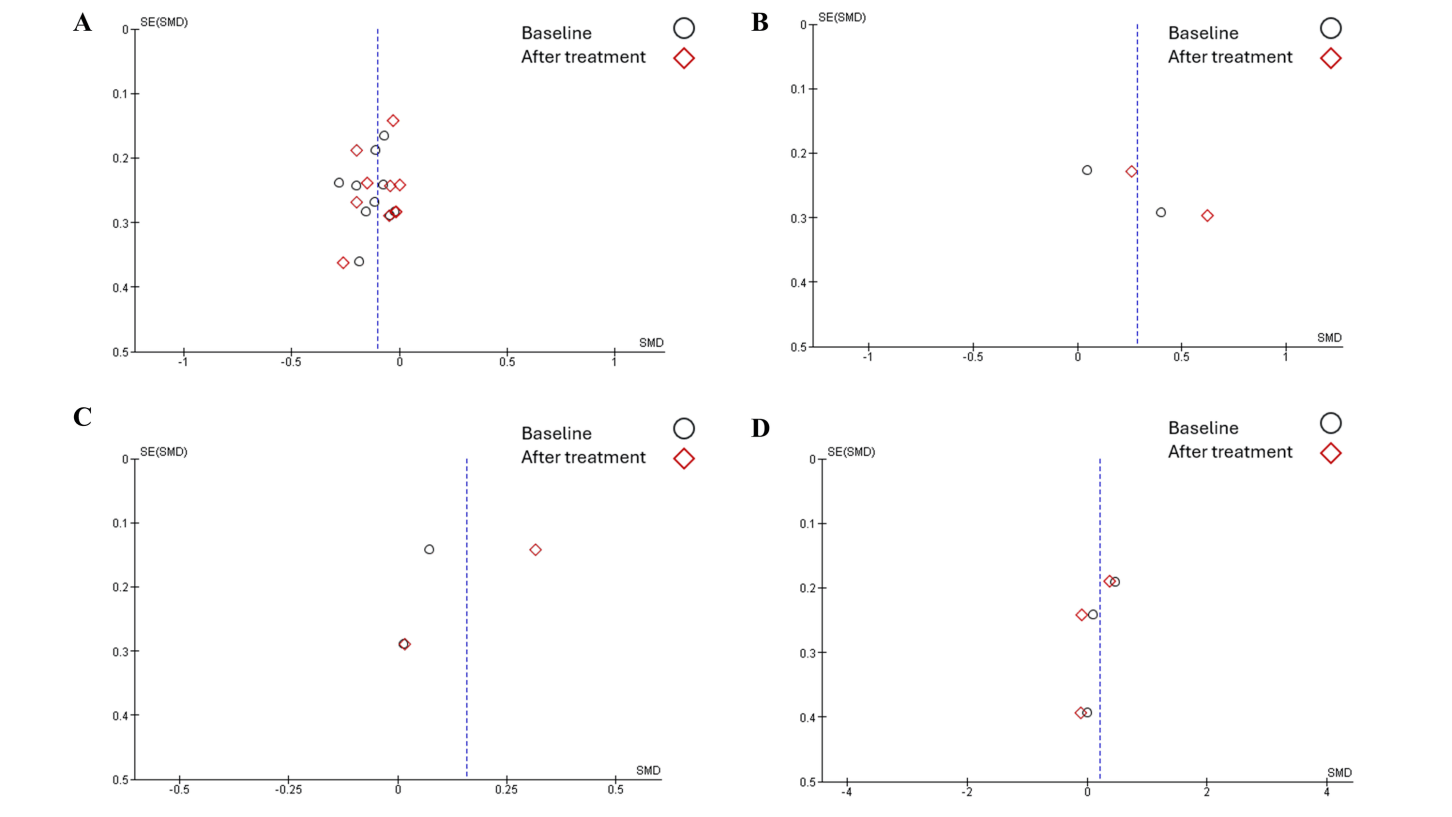
Publication bias among studies - image 1 Studies discussed A) handgrip strength, B) skeletal muscle mass index, C) skeletal muscle mass, and D) total fat mass

**Figure 11:**
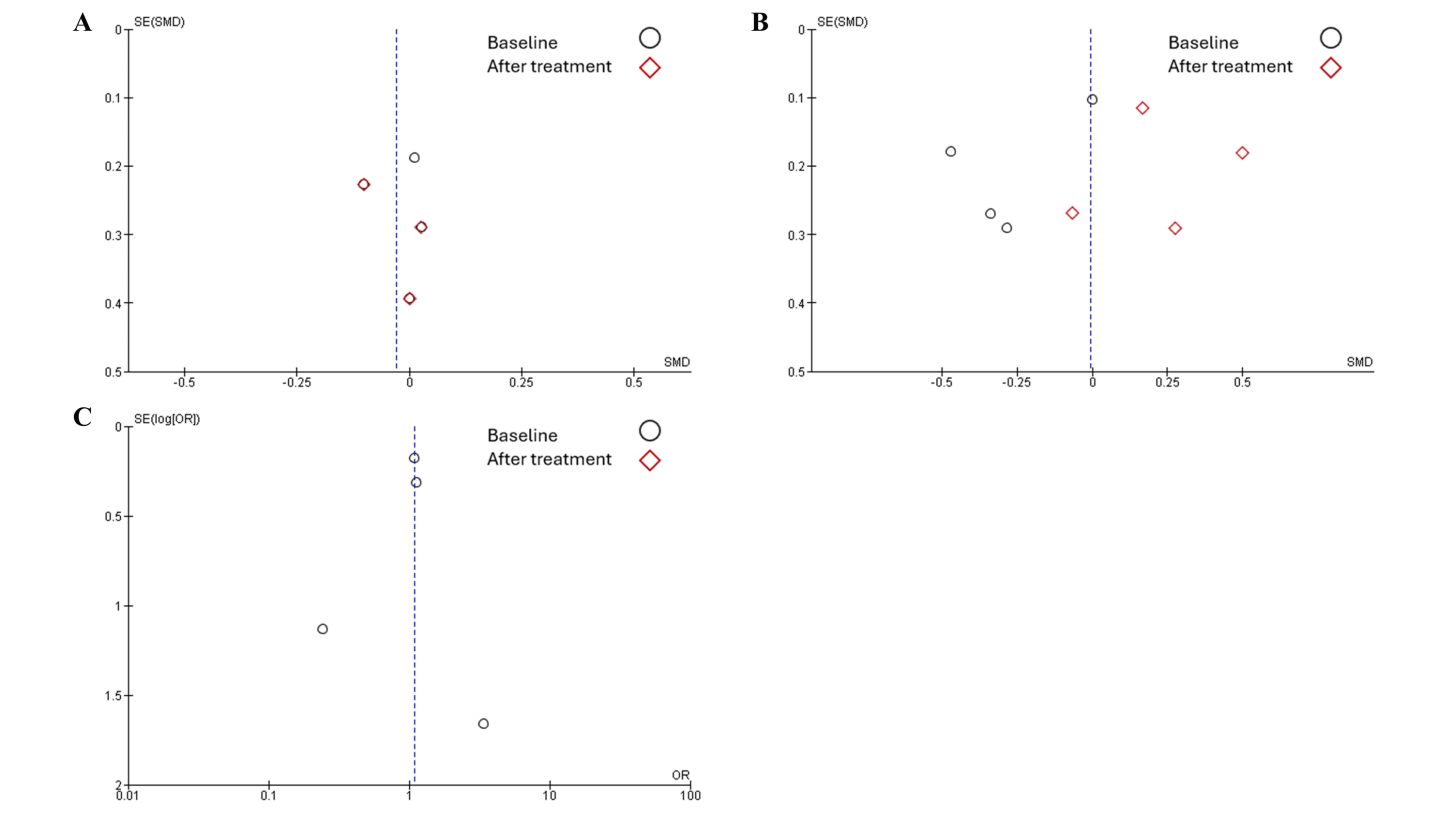
Publication bias among studies - image 2 Studies discussed A) appendicular lean mass, B) gait speed, and C) adverse events

Overall, low publication bias was observed in all studies, as studies were distributed symmetrically and made a clear funnel shape. Furthermore, studies were distributed on both sides of the line (Figures 10, 11).

Certainty of Evidence

Outcomes, like handgrip strength, skeletal muscle mass index, and adverse events, showed a high certainty of evidence, as evidenced by low RoB, low inconsistency, lack of serious indirectness, imprecision, and publication bias. Meanwhile, skeletal muscle mass and appendicular lean mass had moderate certainty of evidence due to 50% of studies having moderate RoB. Meanwhile, gait speed had low certainty of evidence due to a high heterogeneity (serious inconsistency) (Table 4). 

**Table 4:** Certainty of evidence using GRADE assessment framework CI: confidence interval; GRADE: Grading, Reporting, Assessment, Development, and Evaluation; MD: mean difference; OR: odds ratio

Outcomes	Studies	RoB	Inconsistency	Indirectness	Imprecision	Publication bias	Effect size	Certainty of evidence
Handgrip strength (Kg)	9	Low	Not serious (I^2^=0%)	Not serious	Not serious	No	Std. MD: -0.10 (95% CI: -0.21 to 0.00)	High ƟƟƟƟ
Skeletal muscle mass index (Kg/m^2^)	2	Low	Not serious (I^2^=0%)	Not serious	Not serious	No	Std. MD: 0.29 (95% CI: 0.04 to 0.53)	High ƟƟƟƟ
Skeletal muscle mass (Kg)	2	Low-moderate	Not serious (I^2^=0%)	Not serious	Not serious	No	Std. MD: 0.16 (95% CI: -0.02 to 0.33)	Moderate ƟƟƟ
Total fat mass (Kg)	3	Low	Not serious (I^2^=5%)	Not serious	Not serious	No	Std. MD: 0.21 (95% CI: 0.01 to 0.41)	High ƟƟƟƟ
Appendicular lean mass (Kg)	4	Low-moderate	Not serious (I^2^=0%)	Not serious	Not serious	No	Std. MD: -0.03 (95% CI: -0.22 to 0.16)	Moderate ƟƟƟ
Gait speed (m/s)	4	Low	Serious (I^2^=65%)	Not serious	Not serious	No	Std. MD: 0.01 (95% CI: -0.23 to 0.21)	Low ƟƟ
Adverse events	4	Low	Not serious (I^2^=0%)	Not serious	Not serious	No	OR: 1.08 (95% CI: 0.80-1.45)	High ƟƟƟƟ

Discussion

There is an increasing debate over the use of nutritional supplements, which have the potential to be effective for elderly sarcopenia patients, and the type of nutritional supplements that should be used. Thus, this systematic review and meta-analysis assessed the body of evidence on the importance of nutritional supplements in patients with sarcopenia, particularly the elderly, and analyzed the impact of the essential nutrients, such as protein, vitamin D, amino acids, omega-3, creatine, magnesium, zinc, and vitamin B12 in age-related changes associated with sarcopenia, like body composition, muscle mass, strength, physical and functional outcomes. Most of the nutritional interventions identified in the present review used protein supplements, omega 3, vitamin D, amino acids, creatine, vitamin B12, zinc, magnesium, and combination of these nutrients aimed at improving health outcomes, often with or without physical exercise; however, when combining nutritional intervention with physical exercise, it further enhances the clinical outcomes of the patients. Meanwhile, our meta-analysis indicates a significant (p≤0.05) difference in handgrip strength, skeletal muscle mass index, total fat mass, and gait speed (after intervention) between intervention and control groups. However, a non-significant difference (p>0.05) was observed in appendicular lean mass, overall gait speed, and adverse events.

In addition, few studies have reported a significant impact of nutritional supplements on biomarkers (hsCRP, IL-2), which may be attributed to patient characteristics, type of supplements used, and dosage. Overall, our study demonstrated a significant difference because nutritional supplements play an essential role in the maintenance and development of muscle mass and physical function in elderly sarcopenia patients. Moreover, these supplements, particularly whey protein, have a positive impact on the health of their muscles [59]. Additionally, intake of 20-40 g of protein per serving has the potential to maximize the stimulation of synthesis of muscle proteins; however, in case of sarcopenia, due to anabolic resistance, a double dose of whey protein is required [60,61]. A narrative review demonstrated that a higher intake of nutritional supplements increased muscle strength [62]. However, the findings may be limited by the non-inclusion of pooled statistical quantitative data, which can further validate the outcomes.

Similarly, a systematic review with 10 RCTs demonstrated that whey protein significantly increases appendicular muscle mass (std. MD: 0.28, 95% CI: 0.11-0.45), appendicular muscle mass index (std. MD: 0.47, 95% CI: 0.23-0.71), gait speed (std. MD: 1.13, 95% CI: 0.82-1.44), meanwhile reduction was observed in IL-6 concentration (std. MD: -0.32, 95% CI: -0.55 to -0.09) in elder patients with sarcopenia [63]. Likewise, another meta-analysis found non-significant improvement in lean body mass (std. MD: 0.10, 95% CI: -0.14 to 0.34), appendicular skeletal muscle mass (std. MD: 0.15, 95% CI: -0.06 to 0.36), and gait speed (std. MD: 0.17, 95% CI: -0.03 to 0.36); however, there was a significantly increased grip strength (WMD: 1.87, 95% CI: 0.01-3.74) in the intervention group [64]. In contrast, another review observed a non-significant impact of nutritional supplements on handgrip strength (std. MD: 0.36, 95% CI: -0.15 to 0.88), and quadriceps muscle strength (std. MD: 0.11, 95% CI: -0.06 to 0.27), when compared with control [65]. The discrepancy here is that some studies reported that nutritional supplements led to a significant improvement in body composition and physical function, while others, including our study, also observed significant improvement in sarcopenia-associated outcomes. This may be attributed to the improper intake of nutrition; patient characteristics, particularly age, gender, socioeconomic conditions, and nutritional status; and any other comorbidities. Notably, type, dose, and duration of intervention are the other most important indicators of this discrepancy.

Furthermore, whether the intervention is used alone or in combination with physical exercise often enhances the outcomes. Moreover, variability in the assessment methods for measuring body composition and physical function may contribute to this discrepancy. Meanwhile, specialized education programs for nutrition and exercise guidance can enhance and improve the muscle mass and physical functions [66]. Moreover, in the present study, protein and vitamin D were the most common nutrients combined with other nutrients, which indicated that vitamin D addition in every supplement may contribute to the better recovery of physical functions in sarcopenia patients [67]. Another meta-analysis also demonstrated that vitamin D with protein significantly improves handgrip strength (std. MD: 0.38, 95% CI: 0.18-0.47) [68]. Moreover, non-significant and no serious adverse events were reported in the nutritionally supplemented group compared to the control group. Our findings are aligned with the findings of another study, which also observed non-significant differences in adverse events [69]. Likewise, no significant adverse events were associated with the administration of amino acid-based supplements for sarcopenia patients with liver diseases [70].

Key findings of the present study suggest that nutritional supplements can enhance muscle mass and strength. However, it is also recommended to adopt personalized strategies for each patient, keeping in mind the disease severity, comorbidities, and nutritional status, as these indicators can play a major role in deciding which essential nutrients are required. In addition, physical exercise should also be incorporated to minimize the limitations of nutritional therapies.

The present study has several strengths; it comprehensively collected the evidence and presented it quantitatively, with low heterogeneity. In addition, we applied a well-defined GRADE framework for the certainty of evidence. However, it has several limitations as well. For instance, we failed to perform a subgroup analysis for different types of nutrients (protein, vitamin D, amino acids, creatine, omega-3) and nutrition supplements alone and nutrition + physical exercise due to the unavailability of uniform data, and most of the included studies used a combination of these nutrients and exercise. Another limitation is the relatively short intervention duration (12 weeks) in most RCTs. Further multicenter and longitudinal studies are required for the validation of the findings of the present study.

## Conclusions

Our findings show that certain nutritional supplements containing proteins, vitamin D, amino acids, omega-3, creatine, vitamin B12, zinc, magnesium, and other nutrients demonstrated potential in improving muscle strength. Our meta-analysis indicated a significant impact of nutritional supplements on handgrip strength, total fat mass, skeletal muscle mass index, and gait speed (after intervention), while a non-significant impact on appendicular lean mass, gait speed (overall), skeletal muscle mass, and adverse events, compared to the control group. Although supplementation in these domains did not show effectiveness, combining supplementation with physical exercise may further enhance the outcomes. Therefore, using nutritional supplements can be considered as a supportive approach to managing sarcopenia. Future studies should focus on the dosage, given that our findings indicated a lack of significance.

## Appendices

**Table 5:** Literature search strategy employed for different databases MeSH: Medical Subject Headings

Search strategy
PubMed: ("pathophysiology"[All Fields] OR "disease mechanism"[All Fields] OR "disease progression"[MeSH Terms] OR "underlying mechanisms"[All Fields] OR "pathogenesis"[All Fields]) AND ("nutrient"[All Fields] OR "nutrients"[MeSH Terms] OR "diet"[All Fields] OR "diet supplement"[All Fields] OR "Supplementary diet"[All Fields] OR "food supplement"[All Fields] OR "proteins"[MeSH Terms] OR "creatine monohydrate"[All Fields] OR "vitamins"[MeSH Terms] OR "vitamin D"[All Fields] OR "vitamin D3"[All Fields] OR "cholecalciferol"[MeSH Terms] OR "vitamin C"[All Fields] OR "ascorbic acid"[MeSH Terms] OR "vitamin E"[All Fields] OR "vitamin B1"[All Fields] OR "thiamine"[MeSH Terms] OR "vitamin B2"[All Fields] OR "riboflavin"[MeSH Terms] OR "vitamin B6"[All Fields] OR "vitamin b12"[All Fields] OR "omega"[All Fields] OR "magnesium"[MeSH Terms] OR "zinc"[MeSH Terms] OR "lycopene"[MeSH Terms] OR "lutein"[MeSH Terms] OR "zeaxanthins"[MeSH Terms] OR "antioxidants"[MeSH Terms]) AND ("sarcopenia"[MeSH Terms] OR "myopenia"[All Fields] OR "body composition"[All Fields] OR "muscle wasting"[All Fields] OR "muscle loss"[All Fields] OR "skeletal muscle loss"[All Fields] OR "muscular atrophy"[MeSH Terms] OR "muscle decline"[All Fields])
The Cochrane Library: ((“pathophysiology” OR “disease mechanism” OR “disease progression” OR “underlying mechanisms” OR “pathogenesis”)):ti,ab,kw AND ((“nutrient” OR “nutrients” OR “diet” OR “diet supplement” OR “Supplementary diet” OR “food supplement” OR “proteins” OR “creatine monohydrate” OR “vitamins” OR “vitamin D” OR “vitamin D3” OR “cholecalciferol” OR “vitamin C” OR “ascorbic acid” OR “vitamin E” OR “vitamin B1” OR “thiamine” OR “vitamin B2” OR “riboflavin” OR “vitamin B6” OR “vitamin B12” OR “omega” OR “magnesium” OR “zinc” OR “lycopene” OR “lutein” OR “zeaxanthins” OR “antioxidants”)):ti,ab,kw AND ((“sarcopenia” OR “myopenia” OR “body composition” OR “muscle wasting” OR “muscle loss” OR “skeletal muscle loss” OR “muscular atrophy” OR “muscle decline”)):ti,ab,kw
Scopus: (“pathophysiology” OR “disease mechanism” OR “disease progression” OR “underlying mechanisms” OR “pathogenesis”) AND (“nutrient” OR “nutrients” OR “diet” OR “diet supplement” OR “Supplementary diet” OR “food supplement” OR “proteins” OR “creatine monohydrate” OR “vitamins” OR “vitamin D” OR “vitamin D3” OR “cholecalciferol” OR “vitamin C” OR “ascorbic acid” OR “vitamin E” OR “vitamin B1” OR “thiamine” OR “vitamin B2” OR “riboflavin” OR “vitamin B6” OR “vitamin B12” OR “omega” OR “magnesium” OR “zinc” OR “lycopene” OR “lutein” OR “zeaxanthins” OR “antioxidants”) AND (“sarcopenia” OR “myopenia” OR “body composition” OR “muscle wasting” OR “muscle loss” OR “skeletal muscle loss” OR “muscular atrophy” OR “muscle decline”)
ScienceDirect: (“pathophysiology”) AND (“nutrient supplements” OR “proteins” OR “vitamins” OR “magnesium” OR “zinc” OR “lycopene” OR “zeaxanthins”) AND (“sarcopenia”)
Google Scholar: (“pathophysiology” OR “disease progression”) AND (“nutrients” OR “food supplement” OR “proteins” OR “vitamins” OR “magnesium” OR “zinc” OR “lycopene” OR “lutein” OR “zeaxanthins” OR “antioxidants”) AND (“sarcopenia” OR “muscle loss” OR “skeletal muscle loss” OR “muscular atrophy” OR “muscle decline”)
